# In Vitro Metabolic Studies of REV-ERB Agonists SR9009 and SR9011

**DOI:** 10.3390/ijms17101676

**Published:** 2016-10-03

**Authors:** Lore Geldof, Koen Deventer, Kris Roels, Eva Tudela, Peter Van Eenoo

**Affiliations:** Department of Clinical Chemistry, Microbiology and Immunology, Doping Control Laboratory (DoCoLab), Ghent University, Technologiepark 30 B, B-9052 Zwijnaarde, 9000 Ghent, Belgium; Koen.Deventer@UGent.be (K.D.); Kris.Roels@UGent.be (K.R.); eva.tudelapalomar@gmail.com (E.T.); Peter.Vaneenoo@UGent.be (P.V.E.)

**Keywords:** REV-ERB agonists, SR9009, SR9011, phase I metabolism, in vitro studies, liquid chromatography–high resolution mass spectrometry (LC–HRMS), doping agents

## Abstract

SR9009 and SR9011 are attractive as performance-enhancing substances due to their REV-ERB agonist effects and thus circadian rhythm modulation activity. Although no pharmaceutical preparations are available yet, illicit use of SR9009 and SR9011 for doping purposes can be anticipated, especially since SR9009 is marketed in illicit products. Therefore, the aim was to identify potential diagnostic metabolites via in vitro metabolic studies to ensure effective (doping) control. The presence of SR9009 could be demonstrated in a black market product purchased over the Internet. Via human liver microsomal metabolic assays, eight metabolites were detected for SR9009 and fourteen metabolites for SR9011 by liquid chromatography–high resolution mass spectrometry (LC–HRMS). Structure elucidation was performed for all metabolites by LC–HRMS product ion scans in both positive and negative ionization mode. Retrospective data analysis was applied to 1511 doping control samples previously analyzed by a full-scan LC–HRMS screening method to verify the presence of SR9009, SR9011 and their metabolites. So far, the presence of neither the parent compound nor the metabolites could be detected in routine urine samples. However, to further discourage use of these potentially harmful compounds, incorporation of SR9009 and SR9011 into screening methods is highly recommended.

## 1. Introduction

Physiological processes involving metabolism and behavior, e.g., activity/rest, are generally organized on a cycle of approximately 24 h driven by a circadian rhythm [1,2,3]. The nuclear receptors reversed-viral erythroblastosis α and β (REV-ERB α and β) regulate the expression of core clock proteins and therefore help to modulate the circadian rhythm [1,2,4,5].

**Figure 1:**
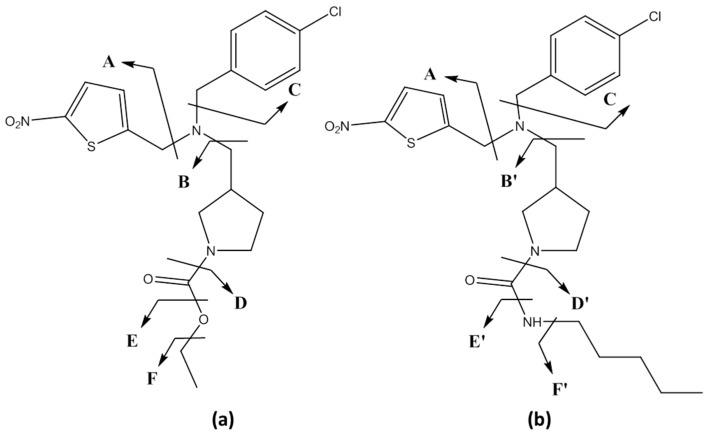
Chemical structures of REV-ERBα agonists SR9009 (**a**) and SR9011 (**b**). The tentative fragmentation patterns are also indicated (by the arrows and the parts of the molecules were assigned to a letter).

Modulation of the REV-ERB activity by synthetic agonists, e.g., SR9009 and SR9011 (Figure 1), alters the expression of genes involved in lipid and glucose metabolism and, therefore, plays an important role in maintaining the energy homeostasis [1,4,6]. Effects of SR9009 and SR9011 observed via in vitro and in vivo animal studies were increased basal oxygen consumption, decreased lipogenesis, cholesterol and bile acid synthesis in the liver, increased mitochondrial content, glucose and fatty acid oxidation in the skeletal muscle and decreased lipid storage in the white adipose tissue [1,2,4,6,7].

The observed increase in energy expenditure and decrease in fat mass make the REV-ERB agonists SR9009 and SR9011 promising drug candidates for the treatment of several metabolic disorders [3,4,6]. At the same time, the increase in exercise capacity observed via in vivo animal studies [6,8] makes these compounds also attractive for performance enhancement by athletes. Such use can be classified as doping. The potential interest as doping agents is clearly shown by their popularity in discussion forums on the Internet, where they are mentioned as the ultimate “exercise in a pill” compounds [8,9,10,11,12,13,14]. Although these REV-ERB agonists are currently still undergoing clinical evaluation and are therefore not approved for therapeutic use, distribution in black market products might be expected as observed before for designer steroids [15,16,17], peptides [18,19,20,21,22,23], several selective androgen receptor modulators (SARMs) [24,25,26] and peroxisome proliferator activated receptor δ (PPARδ) agonists e.g., GW501516 [26].

Even though not explicitly mentioned on the Prohibited List published by the World Anti-Doping Agency (WADA), they are indirectly prohibited as non-approved substances (Class S0), but could potentially also be classified as metabolic modulators (Class S4) [27].

As illicit use of SR9009 and SR9011 can be anticipated, monitoring of their presence on the market and use by doping control laboratories is recommended. These preventive investigations not only help to close the gap between anti-doping laboratories and the appearance of new doping agents, but also contribute to deter the use of these compounds. It would protect fair play and the health of athletes, as athletes are deterred from use when a substance is detectable.

To allow a fast response to the appearance of new non-approved performance-enhancing substances, in vitro metabolic studies are frequently applied. As the liver is the principal organ for drug metabolism, in vitro models are often based on human liver fractions (e.g., human liver microsomes (HLM)) [28]. These in vitro studies not only circumvent the ethical objections related to the use of human volunteers for excretion studies, they are more affordable and can be applied rapidly. Moreover, analytically, the clean extracts improve the characterization of metabolites [29]. However, careful extrapolation of these in vitro studies to real human metabolism should be performed as some metabolic pathways may be over- or under-expressed [28]. Nevertheless, in vitro metabolic studies allow incorporating metabolites into existing screening methods, which could improve the detection window compared to the parent compound, and they can provide reference material to improve the identification of suspicious doping control samples [29].

In the current study, a black market product sold as a performance-enhancing product and labeled to contain SR9009 was purchased over the Internet to verify its content. The analysis resulted in the identification of the mentioned compound by liquid chromatography–(high resolution) mass spectrometry (LC–(HR)MS). Consecutively, HLM were applied to perform metabolic studies of SR9009 and SR9011.

## 2. Results

### 2.1. Analysis of Black Market Product

Reference standards of SR9009 and SR9011 were analyzed by both low resolution and high resolution liquid chromatography–mass spectrometry (LC–(HR)MS) instruments. The presence of SR9009 in the black market product was also verified by comparison with commercially available reference material by LC–(HR)MS. Mass deviations smaller than 1 ppm were observed for SR9009 and SR9011 in the reference material and black market product (LC–HRMS). Identification criteria in chromatography and mass spectrometry, as stipulated in the WADA Technical Document [30], were also met for the black market product. LC–(HR)MS analysis did not result in the detection of major impurities (<5% total peak height in the total ion chromatogram) in the black market product.

### 2.2. In Vitro Metabolic Studies

In the current study, the metabolic fate of SR9009 and SR9011 was determined by HLM incubations with commercially available reference standards. The HLM incubation samples were analyzed by full-scan LC–HRMS analysis in both positive and negative ionization mode. The search for potential metabolites was complemented with the extraction of specific exact mass ions for “expected” metabolites, based on knowledge of HLM transformations and theoretically possible metabolic pathways, including oxidations (e.g., hydroxylations, double bond formation), reductions (e.g., ring opening), cleavage of the structure and combinations of these pathways [31,32].

**Figure 2:**
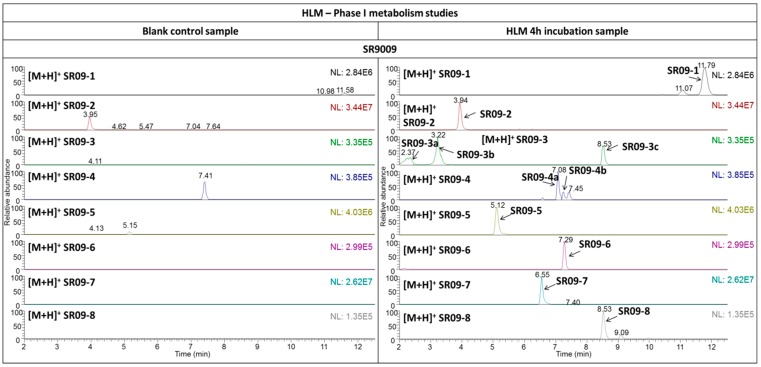
In vitro metabolic studies with SR9009. The extracted ion liquid chromatography–high resolution mass spectrometry (LC–HRMS) chromatograms of the 4-h human liver microsomes (HLM) incubation samples (**right column**) are presented in comparison with blank (without HLM) control samples (**left column**). NL: normalization.

**Figure 3:**
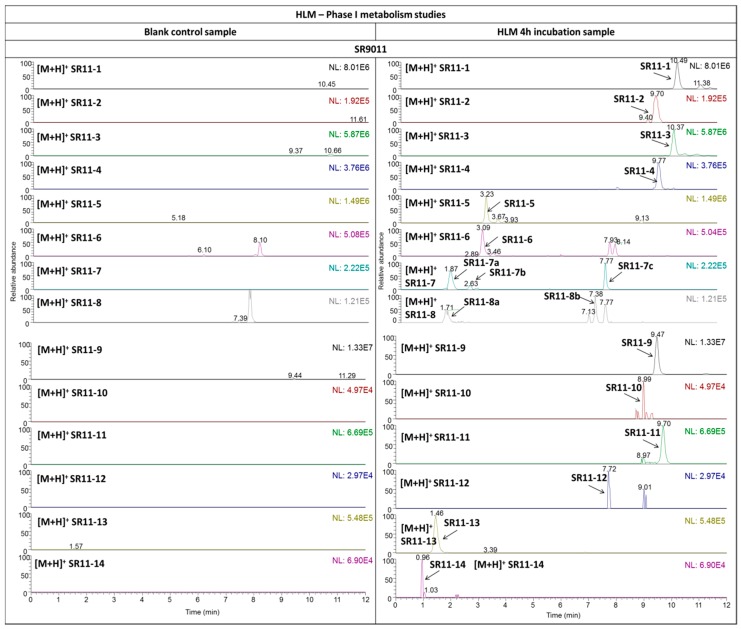
In vitro metabolic studies with SR9011. The extracted ion LC–HRMS chromatograms of the 4-h HLM incubation samples (**right column**) are presented in comparison with blank (without HLM) control samples (**left column**). NL: normalization.

Eight metabolites (SR09-1–SR09-8) and fourteen metabolites (SR11-1–SR11-14) were detected in the HLM incubation samples for SR9009 and SR9011, respectively (Figure 2 and Figure 3). As the metabolites detected in all microsomal incubation samples were very similar, only the results of the 4-h incubation sample are shown in the figures. To facilitate further discussion, the different parts of the molecules of both parent compounds were assigned to a letter (Figure 1 and Table 1). All metabolites were detected with less than a 5-ppm mass deviation from their proposed structures (Table 2 and Table 3).

### 2.3. Structure Characterization by Liquid Chromatography–High Resolution Tandem Mass Spectrometry (LC–HRMS/MS)

**Figure 4:**
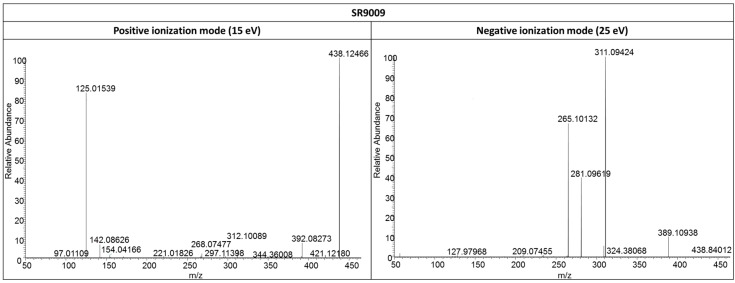
LC–HRMS/MS product ion scan mass spectra of SR9009 in both positive (**left column**) and negative (**right column**) ionization mode at a collision energy (CE) of 15 and 25 eV, respectively.

**Figure 5:**
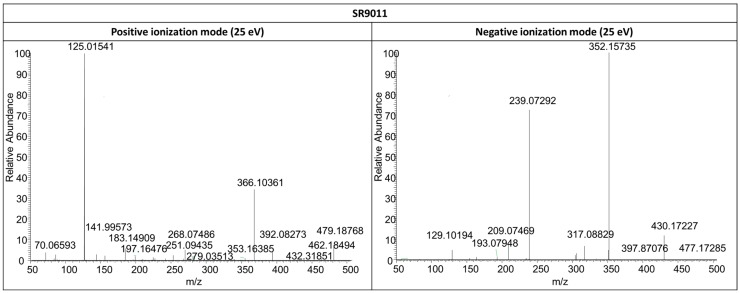
LC–HRMS/MS product ion scan mass spectra of SR9011 in both positive (**left column**) and negative (**right column**) ionization mode at a collision energy (CE) of 25 eV.

Initially, the fragmentation patterns of the parent compounds were studied in both positive and negative ionization mode to identify diagnostic product ions (Figure 1, Figure 4 and Figure 5). Similar fragmentation patterns were observed for SR9009 and SR9011 leading to diagnostic Fragments A, B/B’ and C (Table 1). As indicated in Figure 1, Fragment B/B’ was further fragmented for both SR9009 and SR9011, respectively. Structure elucidation of the SR9009 and SR9011 metabolites was based on the detection of structure-specific product ions by LC–HRMS (Table 2 and Table 3).

#### 2.3.1. SR9009

For all hydroxylated SR9009 metabolites, product ions related to a loss of water were observed (Table 2).

In the product ion scan mass spectrum of SR09-1, a base peak was observed at *m*/*z* 295.0298 in positive ionization mode. This ion was also found for SR9009 and could be linked to C_13_H_12_O_2_N_2_ClS by HRMS (Table 1). Fragments A, B and C were also observed for SR09-1 (Table 1). In negative ionization mode, ions *m*/*z* 327.0895 and 251.0494 indicate a hydroxylation of the parent compound outside C and F (Table 2). In positive ionization mode, a fragment ion *m*/*z* 172.0965 was found, which indicates a hydroxylation in B (Table 2).

The structure of metabolite SR09-2 corresponds to a loss of A of the parent compound. The product ion data were also consistent with this structure, as only Fragments B and C were observed (Table 1 and Table 2). For metabolites SR09-5 and SR09-7, with structures corresponding to the loss of B and D, respectively, only product ions related to A and C were observed (Table 1).

Metabolites SR09-3 and SR09-4 correspond to hydroxylated derivatives of SR09-2 after loss of A (Table 2). For all isomeric SR09-3 and SR09-4 metabolites, Fragment C was observed. In the product ion scan mass spectrum of SR09-3a, *m*/*z* 158.0811 was observed, which indicates a hydroxylation in B (*m*/*z* 142.0863), as this fragment corresponds to C_7_H_12_NO_3_ with a 0.63-ppm mass deviation (Table 2). In contrast to what was observed in the product ion scan mass spectra of all other metabolites, product ion *m*/*z* 154.0418 corresponding to C_8_H_9_NCl was more abundant than product ion *m*/*z* 125.0153 (Fragment C) for SR09-3b. For SR09-3b, *m*/*z* 141.0100 was also observed, which corresponds to C_7_H_6_OCl with 1.12 ppm mass deviation and is indicative for a hydroxylation at C of the parent compound. For metabolites SR09-3c and SR09-4a, no indicative ions for the position of hydroxylation (and further oxidation (–H_2_)) were found. The ion *m*/*z* 170.0364 observed for SR09-4b would indicate a hydroxylation and subsequent keto-formation in C of the parent compound, as this corresponds to C_8_H_9_NOCl with a 1.75-ppm mass deviation (Table 2).

The ion *m*/*z* 157.9904 in the product ion scan mass spectrum of SR09-6 indicates a hydroxylation in A (*m*/*z* 141.9957) for this metabolite (Table 2).

Fragments A and C were observed in the product ion scan mass spectrum of SR09-8. Similar to metabolite SR09-1, an abundant fragment ion *m*/*z* 295.0299 was observed. Although no indicative ions for the position of hydroxylation were found, this base peak (*m*/*z* 295.0299) could also indicate a modification (hydroxylation and keto-formation) in B (outside D) (Table 2).

#### 2.3.2. SR9011

For all SR9011 metabolites, except for SR11-12, the number of losses of water was identical to the proposed number of hydroxylations (Table 3).

Typical product ions of Fragments A, B’ and C were observed for SR11-1, but no indicative ions for the position of hydroxylation were found in positive ionization mode (Table 1 and Table 3). However, in negative ionization mode, ion *m*/*z* 368.1522 indicates a modification outside Fragment C (Table 3).

The presence of ion *m*/*z* 215.1387 in the product ion mass spectrum of metabolite SR11-2 could indicate a dihydroxylation at B’ (*m*/*z* 183.1492) (Table 1 and Table 3).

For SR11-3, no diagnostic product ions for the position of hydroxylation were found in positive ionization mode. However, in negative ionization mode, a product ion *m*/*z* 366.1365 was observed for SR11-3, which is similar to ion *m*/*z* 368.1522 found for SR11-1 (Table 3). This ion indicates a hydroxylation and subsequent keto-formation outside C.

Two product ions (*m*/*z* 227.1387 and 213.1230) were found for SR11-4, which could be indicative for a dihydroxylation, and subsequent keto-formation of one of these hydroxyl groups, in B’.

Besides a loss of water, no diagnostic fragment ions indicating the site of hydroxylation were observed for SR11-5.

Based on the presence of fragment ions *m*/*z* 211.1436 and 197.1280, the proposed site of hydroxylation and subsequent keto-formation of SR11-6 is B’ (Table 3). These product ions correspond to modified fragment ions *m*/*z* 197.1648 and 183.1492, respectively, which were observed for SR9011 (Table 1).

Three isomeric compounds were detected for SR11-7(a/b/c). For both SR11-7a and SR11-7b, a fragment ion *m*/*z* 199.1438 was detected, which is similar to the ion *m*/*z* 197.1280 observed for SR11-6 and indicates also a hydroxylation in B’. For SR11-7c, fragment ions *m*/*z* 229.1782 and 258.0903 indicate a hydroxylation in B’, outside D’.

For SR11-8b, fragment ions *m*/*z* 282.0538 and 256.0745 were observed. This first ion corresponds to a modified ion *m*/*z* 268.0750, which was observed for SR9011. For SR11-12, similar fragment ions (*m*/*z* 282.0537 and 256.0746) and an additional fragment ion *m*/*z* 380.0523 were observed. These fragment ions indicate a similar site of modification (hydroxylation and subsequent keto-formation) for SR11-8b and SR11-12, in particular in A or B’ (outside D’). For SR11-8a, no indicative ions for the site of hydroxylation could be identified.

The observed fragment ions for SR11-9, SR11-11, SR11-13 and SR11-14 were consistent with the proposed structure for these metabolites (Table 3). By comparing the product ion scan mass spectra of metabolites SR11-9 and SR11-11, two indicative ions (*m*/*z* 266.0588 and 249.4784) for the position of the additional modification of SR11-11 could be identified. These indicative ions correspond to fragment ions *m*/*z* 268.0744 and 251.0940 observed for SR11-09 (Table 1). Therefore, the proposed site of double bond formation for SR11-11 is in B’-E’.

The fragment ions *m*/*z* 237.0786 and 194.0729 observed in the product ion scan mass spectrum of SR11-10 in positive ionization mode indicate a hydroxylation at a carbon alpha (of B’ or C) to the central nitrogen.

### 2.4. Assay Validation

The LOD of SR9009 and SR9011, determined by an existing screening method applied by our laboratory for doping control purposes [33], was 2 and 5 ng/mL, respectively. The extraction recoveries were 63% for SR9009 and 56% for SR9011 using a non-optimized, regularly-applied extraction protocol for initial testing of doping control samples (hydrolysis (β-glucuronidase/arylsulfatase, pH 5.2) and liquid–liquid extraction (LLE) (*tert*-butyl methyl ether (TBME), pH 9.5). The matrix effects were determined as ion enhancement of 21% and 20% for SR9009 and SR9011, respectively.

### 2.5. Retrospective Analysis

As SR9009 was purchased via the Internet from a gross sales company, which sells to Internet providers of performance-enhancing drugs, it was clear that the substance may already have been misused by athletes in the period between its introduction on the gross sales market and the completion of our studies. To verify the presence of SR9009, SR9011 and their metabolites in routine doping control samples from that period, retrospective data analysis (data reprocessing) was performed for a three-month period (June 2015–September 2015). Furthermore, the presence of the parent compounds was monitored in our routine screening method for another three months (September 2015–December 2015). For the retrospective data analysis, 1511 samples previously analyzed by our routine full scan LC–HRMS screening method [33] were reprocessed, to verify the presence of the parent compounds and metabolites of SR9009 and SR9011 in these samples. Misuse of SR9009 and SR9011 could however not be demonstrated in our study.

## 3. Discussion

The presence of SR9009 in the black market product indicates that the use of SR9009 is no longer a potential threat, but a real doping threat. Shortly after the purchase of the substance from the gross sale Internet market, the product also appeared for purchase in individual quantities from several Internet suppliers of performance-enhancing substances. This indicates that the substance has now become readily available to athletes and that detection methods need to be developed for the product. Metabolism studies are essential to improve detection (windows) of doping agents. Ideally, human excretion studies can be performed to identify diagnostic metabolites. In case of non-pharmaceutical substances, e.g., SR9009 and SR9011, ethical objections and safety aspects limit the use of human volunteers. Consequently, in vitro metabolic assays (HLM) were applied to perform metabolic studies of SR9009 and SR9011.

**Figure 6:**
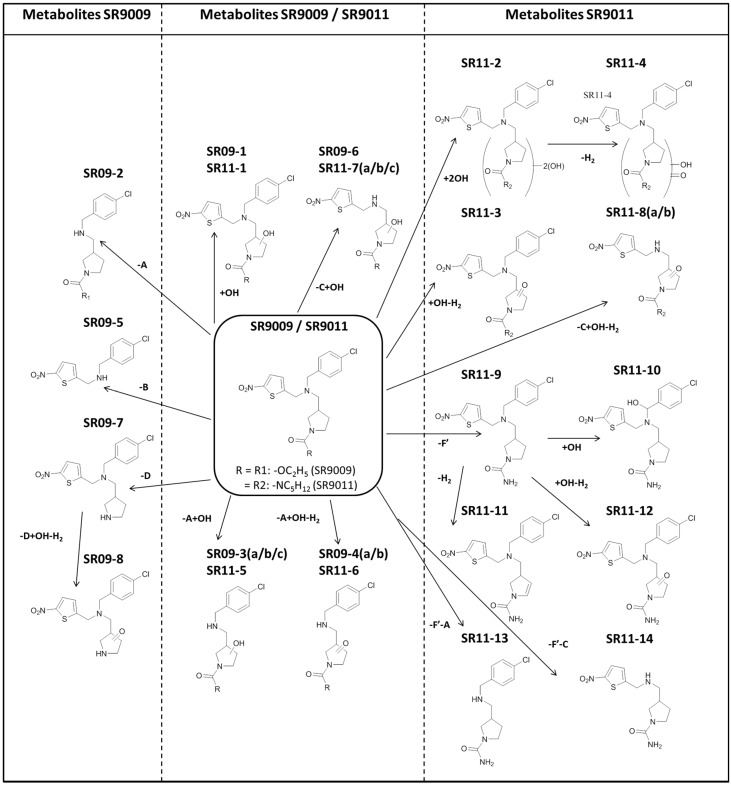
Overview of in vitro metabolic studies with SR9009 and SR9011. The proposed metabolic modifications are also presented; the structures of A, B, C, D and F’ are indicated in Figure 1 and Table 1. For the position of hydroxylations, only one possible configuration is shown.

Eight metabolites (SR09-1–SR09-8) and fourteen metabolites (SR11-1–SR11-14) were detected in vitro for SR9009 and SR9011 by LC–HRMS. For the characterization of the metabolites, application of high resolution instruments can be advantageous, e.g., the metabolic modifications could be verified by determining the mass deviations of the proposed chemical formulas. To further characterize the proposed structures of the metabolites, LC–HRMS product ion scans were performed. Initially, the parent compounds were studied to determine typical, diagnostic ions. The presence of these diagnostic or modified diagnostic fragment ions can enable the identification of the positions of the metabolic modifications. This metabolite identification is time consuming, but HRMS data can also facilitate the correlation of observed product ions to structure specific fragments of the compounds. Tentative structures of these metabolites, based on LC–HRMS product ion scan data, are presented in Figure 6. Although modifications were observed in all fragments (A, B, B’ and C), most modifications occurred in the B/B’ fragment of the parent compounds. However, further research will be needed to unequivocally identify the chemical structures of the metabolites.

Similar metabolites for SR9009 were described in the HLM incubation and human excretion urine samples by Sobolevsky et al. [34]. Metabolites SR09-1 and SR09-6 were not reported in their study. However, two additional metabolites were detected in their study; one metabolite characterized by a loss of D and hydroxylation and another by a loss of C [34]. This latter metabolite was also described as a major human metabolite [34]. Considering the structures of SR09-6 (−C+OH) and SR09-7 (−D), these metabolic modifications observed by Sobolevsky et al. seem possible in our in vitro incubation samples [34].

Sobolevsky et al. also reported similar metabolites for SR9011, but metabolites SR11-2, SR11-3 and SR11-12 were not reported in their study [34]. Two additional metabolites were detected in their study, characterized by loss of C or A [34], with the latter described as a major urinary metabolite [34]. Since hydroxylated metabolites (SR11-5 and SR11-7), after loss of A or C, were detected, these metabolic modifications observed by Sobolevsky et al. seem also possible in our in vitro incubation samples [34].

The highest relative abundances in the HLM incubation samples with SR9009 were observed for metabolites SR09-1, SR09-2, SR09-4(a), SR09-5 and SR09-7. For SR9011, metabolites SR11-1, SR11-3, SR11-5 and SR11-9 have the highest relative abundances in vitro. Therefore, incorporation of metabolites SR09-1, SR09-2, SR09-4(a), SR09-5, SR09-7, SR11-1, SR11-3, SR11-5 and SR11-9 might improve screening for misuse of SR9009 and SR9011. However, it should be noted that this assumption is only based on the relative abundances of the metabolites detected in the HLM incubation samples and that extrapolation from these in vitro studies to the more complex human situation is difficult.

Nevertheless, in the human excretion studies with SR9009 and SR9011 of Sobolevsky et al., metabolites SR09-5 and SR11-5 were indeed described as major metabolites of SR9009 and SR9011, respectively [34]. In addition, metabolites SR11-6 and SR11-13 were described as major human metabolites of SR9011 [34].

The lack of an intact biological system can hamper the extrapolation of in vitro results to the human situation. When applying in vitro techniques, other pharmacokinetic processes, including absorption, distribution and excretion, are not covered. Whereas human administration studies represent a higher complexity, other factors, e.g., phase II metabolism and extrahepatic sites of metabolism, can influence metabolic clearance. Furthermore, HLM consists of enriched drug metabolizing enzymes, which restricts competition for other enzymes and limits the application of HLM for quantitative estimations of specific metabolic pathways.

However, the in vitro approach offers some advantages, such as the production of cleaner and more concentrated extracts, which facilitates the characterization of metabolites, and they allow a fast response to potential new threats. Therefore, HLM can be considered as a valuable ethically acceptable alternative for human metabolic studies of non-pharmaceutical-grade substances.

To circumvent the low extraction recoveries observed when applying a regularly-used sample preparation procedure (enzymatic hydrolysis and LLE at pH 9.5), the combination with a dilute-and-shoot method is recommended.

The presence of SR9009 and SR9011 and their metabolites was verified by retrospective data analysis in 1511 doping control samples. Although misuse of SR9009 and SR9011 could not be demonstrated in our study, it is hoped that other WADA-accredited laboratories will perform a similar retrospective analysis. This would further close the gap between anti-doping laboratories and doped athletes.

## 4. Materials and Methods

### 4.1. Chemicals and Reagents

A black market gross sales product, claiming to contain SR9009, was purchased over the Internet (to prevent athletes from purchasing this product, further details remain confidential). The internal standard (IS) 17α-methyltestosterone was obtained from Organon (OSS, Noord-Brabant, The Netherlands). 4-Hydroxytamoxifen-d5 was purchased from Toronto Research Chemicals (TRC, Toronto, ON, Canada). The reference standard of methandienone was obtained from the National Measurement Institute (NMI, Sydney, North Ryde, Australia). Reference material of SR9009 and SR9011 was purchased from Calbiochem (Merck Chemicals, Nottingham, UK) and Xcess Bio (San Diego, CA, USA), respectively. Pooled HLM from 20–30 donors, the nicotinamide adenine dinucleotide phosphate (NADPH) regenerating system Solutions A and B and phosphate buffer pH 7.4 all from Gentest were purchased from Corning (Amsterdam, The Netherlands). β-Glucuronidase/arylsulfatase from *Helix pomatia* was from Roche Diagnostics (Mannheim, Germany). Ethanol and ammonium acetate (NH_4_OAc) were purchased from Biosolve (Valkenswaard, The Netherlands). Formic acid (HCOOH), ammonium formate (NH_4_OOCH) and methanol (MeOH) were obtained from Fisher Scientific (Loughborough, UK). *tert*-Butyl methyl ether (TBME) was purchased from Macron-Avantor (Deventer, The Netherlands). Sodium acetate (NaOAc), sodium hydrogen carbonate (NaHCO_3_), potassium carbonate (K_2_CO_3_), ammonium iodide (NH_4_I) and acetic acid (HOAc) were from Merck (Darmstadt, Germany). LC-grade water, LC-grade MeOH and LC-grade acetonitrile (ACN) were purchased from J.T. Baker (Deventer, The Netherlands). Nitrogen (N_2_) and oxygen-free nitrogen (OFN) was delivered by Air Liquide (Bornem, Belgium).

### 4.2. Instrumentation

#### Liquid Chromatography–(High Resolution) Mass Spectrometry (LC–(HR)MS)

The same conditions were applied for all liquid chromatography (LC) experiments using a Thermo Finnigan Surveyor Autosampler Plus and an MS Pump Plus (Thermo Scientific, Bremen, Germany). A SunFire™ C18 column (50 mm × 2.1 mm i.d., 3.5 μm) from Waters (AH Etten-Leur, The Netherlands) was applied for the LC separation at a flow rate of 250 μL/min. A volume of 25 μL was injected using the no waste injection mode. LC-grade water (Solvent A) and LC-grade MeOH (Solvent B) both with 1 mM NH_4_OAc and 0.1% HOAc were used as the mobile phase. In the gradient program, the percentage of Solvent B was linearly changed as follows: 0 min, 15%; 1 min, 15%; 6.5 min, 70%; 14 min, 75%; 16.0 min, 100%; 16.9 min, 100%; 17 min, 15% and 20 min, 15%. The total run time was 20 min for the LC methods.

The low resolution experiments were performed using a TSQ Quantum Discovery MAX triple quadrupole mass spectrometer (Thermo Scientific). For these experiments, a full-scan method was applied with a scan range of *m*/*z* 100–500 in both positive and negative mode. The other MS conditions included interface: electrospray ionization (ESI); capillary voltage: 3.5 kV; source temperature: 350 °C; sheath gas (N_2_) pressure: 50 (arbitrary units); auxiliary gas (N_2_) pressure: 20 (arbitrary units); tube lens offset: 100 V; scan time: 0.5 s.

An Exactive mass spectrometer (Thermo Scientific) was applied for the high resolution-tandem mass spectrometry (HR–MS(/MS)) experiments. The instrument operated in a full-scan (both positive and negative) mode with a scan range of *m*/*z* 100–2000 at a resolving power of 50,000 and a data acquisition rate of 2 Hz. LC–HRMS product ion scans were performed by a Q-Exactive mass spectrometer (Thermo Scientific) for the structural investigation of metabolites. Therefore, the protonated and deprotonated molecules were selected as precursor ions with an isolation window of 1.0 *m*/*z* at a resolving power of 70,000, and collision energies of 15, 25, 35 and 45 eV were applied. For both LC–HRMS instruments, the other MS parameters were identical to the low resolution instruments, except for spray voltage: 4 kV, source temperature of 250 °C and heated ESI (HESI) (probe heater at 300 °C). For the assay validation, an LC–HRMS screening method, as indicated in [33], was applied.

### 4.3. In Vitro Incubation Studies

Prior to the in vitro metabolic studies, the black market product containing SR9009 and available reference material of SR9011 and SR9009 were analyzed by LC–(HR)MS for purity verification.

The reaction mixtures for the in vitro metabolic assays (phase I) consisted of 0.1 M phosphate buffer (pH 7.4), a 1.3 mM NADPH regenerating system and the test compound, at a final concentration of 40 μg/mL, dissolved in ethanol (maximum 1% ethanol). The reaction mixtures were first pre-incubated for 5 min at 37 °C using the Eppendorf thermomixer comfort (Rotselaar, Belgium). The enzymatic reactions were then initiated by adding pooled HLM, to obtain a final protein concentration of 0.5 mg/mL. The final samples (250 μL) were incubated at 37 °C for 2, 4 or 18 h. At the appropriate time, the enzymatic reactions were terminated by adding 250 μL of ice-cold MeOH and transferring the tubes into an ice bath for 15 min.

Control samples were used to verify the enzymatic reactions and the stability of the test compounds. These control samples included system blank samples, which did not contain the test compound, and substrate stability samples (blank), which consist of all of the reaction components, except the microsomal proteins. In a positive control sample, the reference standard of methandienone was incubated with HLM.

### 4.4. Assay Validation

The qualitative determination of SR9009 and SR9011 was validated in human urine regarding specificity, extraction recovery and the limit of detection (LOD) according to the Eurachem guidelines [35] and in compliance with the WADA International Standards for Laboratories (ISL) [36].

Specificity was tested during the validation procedure by checking for possible interfering peaks in the extracted ion chromatograms (EIC) at the expected retention times for SR9009 and SR9011.

The LOD was defined as the lowest concentration that can be detected in ten human urine samples with a signal to noise ratio (S/N) higher than three. Ten different blank human urine samples (seven male and three female; pH-range from 4.72–6.89; specific gravity between 1.006 and 1.035) were spiked at 2, 5, 10 and 20 ng/mL for SR9009 and at 2, 5, 10, 20 and 50 ng/mL for SR9011. For the assay validation, 4-hydroxytamoxifen-d5 was used as the internal standard. Blank urines and distilled water samples spiked only with this IS were also included. Sample preparation was performed as described in the following section (4.5. Sample Preparation). The samples were analyzed according to an existing LC–HRMS screening method [33] providing the data necessary to determine the LOD.

The extraction recovery of SR9009 and SR9011 during sample preparation was determined at 20 ng/mL and at 50 ng/mL, respectively. Recovery was calculated by comparison of the mean peak area of the analytes for urine samples spiked before and after sample preparation (hydrolysis/liquid–liquid extraction (LLE), without dilution fraction). Therefore, the results of the ten blank urines applied for the determination of the LOD spiked with SR9009 and SR9011 before sample preparation were used. Another batch of the same blank urines was spiked with SR9009 and SR9011 after sample preparation. Additionally, matrix effects were studied at 20 ng/mL for SR9009 and at 50 ng/mL for SR9011. Matrix effects were measured by comparing the peak area (A) of the analytes spiked in 10 extracted urines and in a neat standard solution following the formula:

$$M.E\;\left(\%\right)=\frac{\left(A\;\left(\mathrm{urine}\right)-A\;\left(\mathrm{standard}\;\mathrm{solution}\right)\right)}{A\left(\mathrm{standard}\;\mathrm{solution}\right)}\;\times \;100$$

### 4.5. Sample Preparation

The samples of the in vitro metabolic assays were analyzed by direct injection on LC–HRMS, after removal of the enzymatic proteins. Briefly, the HLM incubation samples were first centrifuged at 4 °C (12,000× *g*, 5 min) followed by transferring 400 μL into new tubes. Fifty microliters of the internal standard (IS) 17α-methyltestosterone (2 μg/mL) were added to all samples.

The sample preparation procedure for the assay validation and the, 1511 previously analyzed, routine doping control samples was described before [33]. Briefly, a 10-fold dilution fraction of the urine sample (with 1 mM NH_4_OOCH/0.01% HCOOH in H_2_O/ACN (95/5)) was used to reconstitute the dried extract of this urine sample. This extract was obtained after enzymatic hydrolysis (by β-glucuronidase/arylsulfatase from *Helix pomatia* at pH 5.2 and 56 ± 5 °C) and LLE (by TBME at pH 9.5) of the urine sample.

## 5. Conclusions

The presence of SR9009 was demonstrated in a black market product obtained via the Internet. This finding highlights the high threat for the misuse of these potentially performance-enhancing substances and the importance of doping control laboratories to anticipate illicit use of SR9009 and SR9011. HLM incubations led to the detection of eight metabolites for SR9009 and fourteen metabolites for SR9011 by LC–(HR)MS analysis. In this study, the in vitro assays proved to be a valuable tool for the detection and further characterization of SR9009 and SR9011 metabolites.

Although misuse of SR9009 and SR9011 could not be demonstrated, incorporation of these substances into screening methods for doping control purposes can help to discourage the use of these potentially harmful compounds by both amateur and professional athletes.

## Figures and Tables

**Table 1:** Liquid chromatography–high resolution mass spectrometry (LC–HRMS) product ion scans of SR9009 and SR9011. Common product ions of the parent compounds and metabolites are also indicated.

Compound	Fragment ^1^	Chemical Formula	Polarity Mode	Calculated Mass (*m*/*z*)	Δ ppm ^2^	Detected in Metabolites
SR9009/SR9011	A	C_5_H_3_NO_2_S	+	141.9957	2.58	SR09-: 1; 5–8
						SR11-: 1–4; 7a/b/c; 8a/b; 9–11; 14
SR9009	B	C_8_H_11_NO_2_	+	154.0863	0.94	SR09-: 1; 2; 6
SR9011	B’	C_11_H_20_N_2_O	+	197.1648	0.41	/ ^3^
SR9009/SR9011	C	C_7_H_5_Cl	+	125.0153	3.96	SR09-: 1; 2; 3(a/b/c); 4(a/b); 5; 7; 8 SR11-: 1–6; 9–13
SR9009	−C	C_13_H_17_N_3_O_4_S	+	312.1013	1.78	SR09-1
			−	311.0945	1.30	/ ^3^
	−C−NO_2_	C_13_H_18_N_2_O_2_S	−	265.1016	1.25	/ ^3^
SR9011	−C	C_16_H_24_N_4_O_3_S	+	353.1642	1.99	SR11-: 1; 7
	−C−D’	C_10_H_13_N_3_OS	−	239.0734	1.86	SR11-: 1; 3; 9
	−C−D’−NO_2_	C_10_H_14_N_2_S	−	193.0805	4.83	SR11-: 1; 9
	−D’+2H	C_17_H_20_N_3_O_2_ClS	+	366.1038	2.18	SR11-: 1–4; 9
SR9009/SR9011	−E/E’	C_18_H_18_N_3_O_3_ClS	+	392.0830	2.08	SR11-: 1–4
SR9009/SR9011	C+CNH_4_	C_8_H_8_NCl	+	154.0418	1.06	SR09-: 3(b/c); 4b; 8
						SR11-: 1–6; 9–11; 13
SR9009	B−CH_2_	C_7_H_11_NO_2_	+	142.0863	0.18	SR09-: 2; 3(b/c); 4b
	−B+CH_2_	C_13_H_11_N_2_O_2_ClS	+	295.0303	0.96	SR09-: 1; 8
						SR11-: 10; 11
	−A	C_15_H_19_N_2_O_2_Cl	+	295.1208	0.72	SR09-: 1; 3a/b/c
SR9011	B’−CH_2_	C_10_H_18_N_2_O	+	183.1492	0.49	SR11-: 8b
SR9009/SR9011	−C−E/E’	C_11_H_13_N_3_O_3_S	+	268.0750	0.12	SR11-: 1, 2; 4; 7a/b; 8a; 9; 14

^1^ See Figure 1 for the structures of Fragments A, B, B’, C, D, D’, E and E’; − in the fragment column: symbolizes the loss of the indicated fragment; ^2^ Δ ppm: mass deviation between calculated and experimental mass; ^3^ /: this fragment was not observed in metabolites.

**Table 2:** Characterization of metabolites detected in HLM incubation samples with SR9009 by LC–HRMS analysis.

Compound	Metabolic Modification ^1^	Chemical Formula	[M + H]^+^ (∆ ppm)	Product Ion				
				Polarity Mode	Exp *m*/*z* ^2^	Chemical Formula	∆ ppm ^3^	Origin ^1^
SR09-1	+OH	C_20_H_24_N_3_O_5_ClS	454.1192 (0.94)	+	172.0965	C_8_H_14_NO_3_	1.68	B+H_2_O
				−	327.0895	C_13_H_17_N_3_O_5_S	0.31	−C+OH
					281.0476	C_11_H_11_N_3_O_4_S	0.09	−C−F+OH
SR09-2	−A	C_15_H_21_N_2_O_2_Cl	297.1361 (1.08)	+	251.0942	C_13_H_16_N_2_OCl	1.42	−A−E
SR09-3 a	−A+OH	C_15_H_21_N_2_O_3_Cl	313.1309 (1.36)	+	251.0942	C_13_H_16_N_2_OCl	1.38	−A−E−H_2_O
					225.1150	C_12_H_18_N_2_Cl	1.30	−A−D−H_2_O
					158.0811	C_7_H_12_NO_3_	0.63	B+CH_2_+OH
SR09-3 b					141.0100	C_7_H_6_OCl	1.12	C+OH
SR09-3 c					267.0890	C_13_H_16_N_2_O_2_Cl	1.95	−A−F−H_2_O
SR09-4 a	−A+OH−H_2_	C_15_H_19_N_2_O_3_Cl	311.1148 (1.19)	+	265.0731	C_13_H_14_N_2_O_2_Cl	0.85	−F−H_2_O
SR09-4 b					293.1046	C_15_H_18_N_2_O_2_Cl	1.88	−H_2_O
					265.0734	C_13_H_14_N_2_O_2_Cl	1.78	−A−F−H_2_−H_2_O
					170.0364	C_8_H_9_NOCl	1.75	A+CH_3_NO
SR09-5	−B	C_12_H_11_N_2_O_2_SCl	283.0297 (1.88)	+	see ^4^			
SR09-6	−C+OH	C_13_H_19_N_3_O_5_S	330.1112 (1.84)	+	284.0694	C_11_H_14_N_3_O_4_S	2.05	−C−F−H_2_O
					157.9904	C_5_H_4_NO_3_S	1.33	A+OH
SR09-7	−D	C_17_H_20_N_3_O_2_ClS	366.1031 (1.81)	+	see ^4^			
SR09-8	−D+OH−H_2_	C_17_H_18_N_3_O_3_ClS	380.0826 (1.15)	+	362.07120	C_17_H_17_N_3_O_2_ClS	1.36	−H_2_O

^1^ The structures of A, B, C, D and F’ are indicated in Figure 1 and Table 1; − in the columns of metabolic modification and origin: symbolizes the loss of the indicated fragment; ^2^ Exp *m*/*z* = experimental *m*/*z*; ^3^ Δ ppm: mass deviation between calculated and experimental mass; ^4^ see product ion scan data of the parent compounds presented in Table 1 for typical fragment ions of the compound(s).

**Table 3:** Characterization of metabolites detected in HLM incubation samples with SR9011 by LC–HRMS analysis.

Compound	Metabolic Modification ^1^	Chemical Formula	[M + H]^+^ (∆ ppm)	Product Ion				
				Polarity Mode	Exp *m*/*z* ^2^	Chemical Formula	∆ ppm ^3^	Origin ^1^
SR11-1	+OH	C_23_H_31_N_4_O_4_ClS	495.1818 (1.84)	+	181.1333	C_10_H_17_N_2_O	1.49	B’−CH_2_−H_2_
				−	368.1523	C_16_H_24_N_4_O_4_S	0.34	−C+OH
SR11-2	+2OH	C_23_H_31_N_4_O_5_SCl	511.1771 (1.09)	+	242.0954	C_10_H_16_N_3_O_2_S	1.67	−C−D’−2H_2_O
					215.1387	C_10_H_19_N_2_O_3_	1.53	B’−CH_2_−H_2_+2OH
SR11-3	−H_2_+OH	C_23_H_29_N_4_O_4_ClS	493.1665 (1.24)	+	475.1562	C_23_H_28_N_4_O_3_ClS	0.62	−H_2_O
				−	366.1365	C_16_H_22_N_4_O_4_S	0.69	−C+OH−H_2_
SR11-4	−H_2_+2OH	C_23_H_29_N_4_O_5_ClS	509.1616 (0.80)	+	409.1091	C_18_H_22_N_4_O_3_ClS	1.06	−F’−2H_2_O
					242.0952	C_10_H_16_N_3_O_2_S	2.33	−C−D’−2H_2_O
					227.1387	C_11_H_19_N_2_O_3_	1.45	B’+2OH−H_2_
					213.1230	C_10_H_17_N_2_O_3_	1.59	B’−CH_2_+2OH
SR11-5	−A+OH	C_18_H_28_N_3_O_2_Cl	354.1932 (3.11)	+	225.1147	C_12_H_18_N_2_Cl	2.81	−A−D’−H_2_O
					197.1489	C_11_H_19_N_2_O	1.28	B’
					144.1130	C_6_H_14_N_3_O	1.24	B’−F’
SR11-6	−A+OH−H_2_	C_18_H_26_N_3_O_2_Cl	352.1781 (1.59)	+	225.1149	C_12_H_18_N_2_Cl	1.61	−A−D’−H_2_O
					211.1436	C_11_H_19_N_2_O_2_	2.29	B’+OH-H_2_
					197.1280	C_10_H_17_N_2_O_2_	2.20	B’−CH_2_ +OH−H_2_
SR11-7 a/b	−C+OH	C_16_H_26_N_4_O_4_S	371.1742 (1.52)	+	242.0953	C_10_H_16_N_3_O_2_S	2.00	−C−D’−H_2_O
					199.1438	C_10_H_19_O_2_N_2_	1.63	B’−CH_2_+OH
SR11-7 c					258.0903	C_10_H_16_N_3_O_3_S	1.51	−C−D’+OH
					229.1782	C_11_H_23_N_3_O_2_	1.30	B’+OH
SR11-8 a	−C+OH−H_2_	C_16_H_24_N_4_O_4_S	369.1587 (1.20)	+	285.1011	C_11_H_17_N_4_O_3_S	1.47	−C−F’−H_2_O
					242.0953	C_10_H_16_N_3_O_2_S	1.67	−C−D’−H_2_O
SR11-8 b					282.0538	C_11_H_12_N_3_O_4_S	1.89	−C−E’+OH
					256.0745	C_10_H_14_N_3_O_3_S	2.26	−C−D’+OH
SR11-9	−F’	C_18_H_21_N_4_O_3_ClS	409.1088 (1.97)	+	283.0853	C_11_H_15_N_4_O_3_S	2.15	−F’−C
					251.0940	C_13_H_16_N_2_OCl	2.90	−A−E’
SR11-10	−F’+OH	C_18_H_21_N_4_O_4_ClS	425.1051 (1.39)	+	364.0876	C_17_H_18_N_3_O_2_ClS	1.41	−D’−H_2_O
					237.0786	C_12_H_14_N_2_OCl	1.17	−A−D’+OH
					194.0729	C_6_H_14_N_2_O_3_S	4.92	A+CH_2_+OH
SR11-11	−F’−H_2_	C_18_H_19_N_4_O_3_ClS	407.0932 (1.83)	+	266.0588	C_11_H_12_N_3_O_3_S	2.06	−C−E’−H_2_
					249.0784	C_13_H_14_N_2_OCl	2.20	−A−E’−H_2_
SR11-12	−F’−H_2_+OH	C_18_H_19_N_4_O_4_ClS	423.1621 (1.94)	+	380.0823	C_17_H_19_N_3_O_3_ClS	1.86	−D’+OH
					282.0537	C_11_H_12_N_3_O_4_S	2.21	−C−E’+OH
					256.0746	C_10_H_14_N_3_O_3_S	2.03	−C−D’+OH
SR11-13	−F’−A	C_13_H_18_N_3_OCl	268.1205 (2.15)	+	225.1149	C_12_H_18_N_2_Cl	1.66	−A−D’
SR11-14	−F’−C	C_11_H_16_N_4_O_3_S	285.1011 (1.57)	+	see ^4^			

^1^ The structures of A, B, C, D and F’ are indicated in Figure 1 and Table 1; − in the columns of metabolic modification and origin: symbolizes the loss of the indicated fragment; ^2^ Exp *m*/*z* = experimental *m*/*z*; ^3^ Δ ppm: mass deviation between calculated and experimental mass; ^4^ see product ion scan data of the parent compounds presented in Table 1 for typical fragment ions of the compound.
